# Editorial: Alcohol and energy drinks: is this a really good mix?

**DOI:** 10.3389/fnbeh.2023.1213723

**Published:** 2023-05-31

**Authors:** Elio Acquas, Laura Dazzi, Mercè Correa, John D. Salamone, Valentina Bassareo

**Affiliations:** ^1^Department of Life and Environmental Sciences, University of Cagliari, Monserrato, Italy; ^2^Department of Psychobiology, University Jaume I, Castelló, Spain; ^3^Department of Psychological Sciences, University of Connecticut, Storrs, CT, United States; ^4^Department of Biomedical Sciences, University of Cagliari, Monserrato, Italy

**Keywords:** energy drinks, alcohol, caffeine, taurine, brain development, AMED, adolescence

From the first appearance of Red Bull (RB) on the market in 1987 (Reissig et al., 2009), energy drinks (EDs) have become increasingly popular among adolescents and young adults, and their consumption has increased to an alarming extent. There are some well-known adverse effects on the cardiovascular and cerebrovascular systems, such as cardiomyopathies, atrial and ventricular arrhythmias, myocardial infarctions, cardiac death (Mangi et al., 2017; Ehlers et al., 2019; Cao et al., 2021; Piccioni et al., 2021) and cardiovascular and cerebrovascular modifications (Costa et al., 2023). Other effects have also been reported including excitability, irritability, anxiety, insomnia, headache, malaise, nausea/vomiting and abdominal pain (Wolk et al., 2012; Nadeem et al., 2021; Khouja et al., 2022). Moreover, it has been described that the intake of great amounts of EDs produces convulsions, cerebral hemorrhage, psychosis, suicidal thoughts, and acute liver and renal damage (Hernandez-Huerta et al., 2017; Kim et al., 2018; Kelsey et al., 2019).

The articles collected in this Frontiers Research Topic provide an overview of the results of recent studies on the effects of EDs in rodents, the role of some of the common ingredients of EDs on alcohol-induced behaviors, the side effects of the use of alcohol mixed with ED (AMED) for the developing brain of adolescents and the medical and socio-legal standpoints on this EDs association.

Petribu et al. analyzed several studies reporting that the acute or chronic AMED consumption, enhanced the alcohol intake and his motivational value, dose-dependently stimulated locomotion, and deteriorated movements capability. AMED intake time-dependently reduced anxiety, and a wide variety of effects on memory have been reported. The acute administration of AMED did not modify alcohol metabolism, but after its chronic assumption, blood alcohol concentrations were higher or lower than those of the alcohol-only group depending on the paradigm used. A comparable range of effects has been described about metabolic dysfunction. AMED intake produced an increase of pro-inflammatory cytokines, created oxidative stress and lipid peroxidation, but also in this case the results obtained depend on the protocol used. Briefly, AMED produces different outcomes depending on the amount of alcohol and EDs and the age, sex, and line of animals used.

Unfortunately, different studies have been conducted on EDs that contain several substances, but fewer studies have focused on the individual ingredients at well-established concentrations. This is a critical point that may preclude the identification of any potential interaction between components. Thus, while the association between caffeine, one of the principal ingredients of EDs, and alcohol has been previously analyzed, the associations with other active ingredients such as taurine, glucuronolactone, sucrose, B vitamins, are less studied. Sefen et al. addressed this issue and reported that the stimulatory properties of caffeine reduce the sedative effects of alcohol, making individuals more inclined to consume higher amount of alcohol -as they do not perceive the feeling of drunkness- and promoting risk-taking behavior. The increase of AMED drinking can be explained through activation on adenosine A_2A_ receptors, which reduces alcohol intake (Houchi et al., 2008). Moreover, caffeine-induced inhibition of this receptor may contribute to enhanced alcohol consumption (Ferré, 2010). As reported by Cadoni and Peana, preclinical studies using rats, described that caffeine can promote alcohol self-administration (Kunin et al., 2000; Arolfo et al., 2004). Moreover, Sefen et al. indicated that caffeine significantly prevented alcohol-elicited conditioned-place preference and the acquisition of alcohol-elicited conditioned-place aversion (Porru et al., 2020). This seems to suggest that caffeine may have a protective effect on the abuse potential of alcohol. However, Cadoni and Peana referred that Vargiu et al. (2021) found that chronic consumption of RB in adolescent rats potentiates dopamine transmission in the nucleus accumbens shell by a nonadaptive mechanism, like drugs of abuse. The repeated stimulation of dopamine in the nucleus accumbens shell after repetitive EDs consumption can underlie the gateway effect toward the use of different substances of abuse.

Another relevant component of EDs is taurine, the most abundant intracellular amino acid in humans. Taurine does not affect levels of dopamine (Wu et al., 2017), but this compound can modify dopaminergic transmission in brain circuits involved in the regulation of drug intake (Adermark et al., 2011; Chen et al., 2018). Pulcinelli et al. (2020) reported that chronic administration of taurine enhances the voluntary intake of alcohol; they hypothesized that this result could be explained by an anxiolytic effect of taurine obtained by an interaction between GABA and dopamine systems.

The amount of B2, B3, B5, B6, and B12 vitamins in several EDs is higher than the recommended daily doses (Jagim et al., 2022), and the only evidence referred by Tarragon suggests that a proper intake of vitamins with the diet might limit the adverse consequences of alcohol on B-vitamin metabolism (Miyazaki et al., 2012).

An important aspect highlighted by Cadoni and Peana is the serious concern on the negative effects of EDs intake on the processes of neurodevelopment in the brain of adolescents that are the main consumers of these beverages (Seifert et al., 2011; Gallimberti et al., 2013).

Purines are involved in the refinement of several processes during the development of the brain, in particular in its growing architecture (Rodrigues et al., 2019). Acting on A_1_ receptors in different brain areas (hippocampus and cortex), adenosine modulates immature synapses (Jeong et al., 2003; Kerr et al., 2013; Burnstock and Dale, 2015). Accordingly, an excessive intake of caffeine can interfere with the purinergic transmission resulting in altered brain development that can lead to alterations of different brain functions (Silva et al., 2013; Al-Basher et al., 2018; Serdar et al., 2019; Zhang et al., 2020, 2022; Christensen et al., 2021; Agarwal et al., 2022). In addition, Brown et al. (2020) described that an excessive intake of taurine by adolescents or young adults can impair learning and memory function. The suggested mechanism for many alterations in several brain functions is an impairment of oligodendrocytes and neurons development, and of the harmonic process of synaptic pruning and formation of new synapses during the *final cortical maturation* (Serdar et al., 2019).

Finally, the original report by Sefen et al. on the regulation of AMED around the world can help the scientific community and influence public opinion and regulatory agencies to reflect on the need to regulate the sale and the consumption of EDs and AMED. In this regard, we foresee that the articles presented in this Research Topic provide a view of current preclinical and clinical studies in the field and allow new insights into the effects of AMED consumption on human health.

## Author contributions

VB wrote the article. EA, LD, MC, and JS critically revised and approved the final version of the manuscript. All authors contributed to the article and approved the submitted version.
